# Predictive value of Leukocyte ImmunoTest (LIT™) in cancer patients: a prospective cohort study

**DOI:** 10.3389/fonc.2022.897968

**Published:** 2022-08-01

**Authors:** Xiaomeng Li, Xiaojun Ma, Yifeng Liu, Enqiang Chang, Jiang Cui, Daqing Ma, Jiaqiang Zhang

**Affiliations:** ^1^ Division of Anaesthetics, Pain Medicine and Intensive Care, Division of Surgery, Department of Surgery and Cancer, Faculty of Medicine, Imperial College London, Chelsea and Westminster Hospital, London, United Kingdom; ^2^ Department of Anaesthesiology and Perioperative Medicine, Henan Provincial People’s Hospital, People’s Hospital of Zhengzhou University, Zhengzhou, China; ^3^ Research Department of Primary Care and Population Health, University College London, London, United Kingdom

**Keywords:** cancer diagnosis, immune function, Leukocyte ImmunoTest, predictive value, cohort study

## Abstract

Early diagnosis of cancer is crucial to initiate prompt treatment for better patient outcomes. The host immune function and its associated modulators are considered to be potential biomarkers for early cancer diagnosis. Immune and immune-checkpoint biomarkers have been reported to contribute to cancer development, while a high neutrophil-to-lymphocyte ratio has been shown to be associated with poor survival outcomes in a variety of cancers. One hundred sixty-one cancer patients were recruited to take a cost-effective novel Leukocyte ImmuneTest (LIT). LIT was measured to objectively determine the pre-treatment immune status of patients. The correlation between LIT and other conventional diagnostic markers or tumor-related variables was then investigated. Significant correlations between LIT and white blood cell count, smoking status, and tumor stage 4 were found. In addition, the LIT score significantly differentiated between malignant and benign tumors in this study population. Our work raises the possibility to use LIT for general screening surveillance before further costly specialized equipment is applied for cancer diagnosis.

## Introduction

Cancer remains a leading cause of death worldwide. More than 19 million new cancer cases were reported in 2020, and cancer caused 10 million deaths in 2020 globally (1). In general, most cancers are curable through appropriate early screening. Immune-related biomarkers are commonly applied in cancer diagnosis, prognosis, and treatment monitoring. For instance, a high neutrophil-to-lymphocyte ratio has been reported to be associated with poor survival outcomes in a variety of cancers (2–5). However, these measurements often require multiple laboratory procedures including centrifugation to extract certain cells from whole blood or plating cells on glass slides for cell activation. Unfortunately, those procedures may disrupt the natural presentation of immune cells and trigger false cell activation or damage natural cell–cell interaction in the circulation (6). Other inflammatory markers for cancer diagnosis are erythrocyte sedimentation rate (ESR), C-reactive protein (CRP), procalcitonin (PCT), and D-dimer. Nevertheless, the poor sensitivity of these inflammatory markers hinders their ability to be an independent predictor of cancer (7). Therefore, affordable, simple, and easily accessed immune-related tests are needed for the diagnosis and prognosis of cancer.

An innovative technique termed Leukocyte ImmunoTest™ (LIT™, Seroxo Ltd., UK), also known as the leukocyte coping capacity test, has been developed to quantify the reactivity of leukocytes (primarily neutrophils) in response to phorbol 12-myristate 13-acetate (PMA) stimulation (8). The amount of reactive oxygen species (ROS) produced by circulating leukocytes during the PMA challenge is measured through a real-time chemiluminescent measurement (9, 10). This technique has been previously used to assess subjective mental workload (9) and physiological stress levels (10) of healthy volunteers when given various stress challenges. A recent study has demonstrated the LIT assay to be a novel biomarker of neutrophil function (11). However, its other applications including the predictive value of cancer diagnosis or prognosis have not been established. The objective of this study was to investigate the pre-treatment immune status of cancer patients with LIT and to correlate LIT with other cancer diagnostic approaches to evaluate its cancer predicting potential.

## Methods

### Patient enrollment

After this prospective cohort study was approved by the Ethics Committee of Henan Provincial People’s Hospital, Zhengzhou University, Zhengzhou, China (ethics approval no. 201944) and written informed consent was obtained, 161 patients were enrolled from June 2019 to October 2020. Among all participants, 82 were advanced malignant cancer patients (cancer group), and 79 were benign tumor patients (control group). The inclusion criteria were a) diagnosed with a certain type of cancer by pathologic examination (histology or cytology examination) and b) no chemotherapeutics, surgery, or medication before enrolment. Exclusion criteria were psychological or psychiatric illness including anxiety and depression, ongoing infection, a history of autoimmune diseases, or receiving immunomodulation treatment.

### Data collection

#### Clinical and laboratory characteristics

Patients’ demographic data including age, gender, history of smoking, and body mass index (BMI) were collected before any diagnostic tests. According to the standard procedures in the hospital, patients were then referred to undertake a series of laboratory tests and data such as red blood cell (RBC) count, white blood cell (WBC) count, hemoglobin level, platelets level, absolute neutrophil count (ANC), absolute lymphocyte count (ALC), and neutrophil-to-lymphocyte ratio (NLR) were collected. Laboratory results include inflammatory markers such as ESR, CRP, PCT, and D-dimer and tumor markers such as carcinoembryonic antigen (CEA), alpha-fetoprotein (AFP), free prostate-specific antigen (F-PSA), total prostate-specific antigen (T-PSA), and human chorionic gonadotropin (HCG) were obtained.

#### Leukocyte ImmunoTest

ROS production was measured according to the method previously described (6, 8–10). Briefly, 10-μl samples of fresh blood (through finger prick using sterile single-use Unistik lancets) were added to 100 μl of phosphate-buffered saline containing PMA (Sigma, UK) and luminol. The solution was incubated for 10 min at 37.5°C in an Accublock heater block (Labnet, New Jersey, USA). Chemiluminescence was quantified after 10 min using a 3M handheld luminometer (Clean-Trace, NG3) in relative light units. Triplicate LIT scores for neutrophil function were calculated for individual patients to minimize any human error, and the average of triplicate LIT readings was recorded.

#### Tumor-related data

Tumor samples removed by either surgery or biopsy were histologically examined to determine if tumors were malignant or benign. Cancer-related data such as cancer type, tumor-node-metastases (TNM) stage, and maximum cancer core length (MCCL) measured *via* computed tomography scan, ultrasound, magnetic resonance imaging, positron emission tomography scan, and X-rays were collected with the hospital’s standard patient care protocols.

### Statistical analysis

Continuous variables such as patients’ age, blood test results, tumor size, and LIT scores were presented as mean ± SD or median (range), where appropriate. Categorical variables such as gender, history of smoking, TNM stage, and tumor type were presented as numbers (%). A Welch’s t-test was performed to compare mean LIT between benign and malignant cohorts. A linear model using an ordinary least-square estimator was fitted for mean LIT. Comparison of other variables between the two cohorts was carried out by both univariate and multivariate analyses, and model fit was compared by the R^2^. For variables in which there were more than 3% missing data, multiple imputations were carried out for sensitivity analysis. Pearson’s correlation test was performed to find out the correlation between mean LIT and continuous variables, and point biserial correlation was performed to find out the correlation between mean LIT and categorized variables. A receiver operating characteristic (ROC) curve with a cutoff value was generated to illustrate the sensitivity and specificity of LIT in diagnosing malignant tumors. Positive predictive value and negative predictive value were calculated based on the sensitivity, specificity of the ROC curve, and total patient number. The sensitivity and specificity of conventional diagnostic markers were also analyzed with the ROC curve. R software version 3.6.3 (R Foundation for Statistical Computing, Vienna, Austria) and Prism 6.0 (GraphPad, US) were used for statistical analysis.

## Results

### Patients’ characteristics and Leukocyte ImmunoTest score

In total, 79 (49%) patients with benign tumors (control group) and 82 (51%) patients with malignant tumors (cancer group) were recruited for this study. Patients’ demographics and laboratory data with their LIT are presented in **Table 1**, and patients’ tumor-related data are shown in **Table 2**. The mean age of our analytic sample was 46 with 93.6% of them female, and only 5% were smokers. No statistical difference in blood tests, inflammatory markers, and tumor markers was found between benign and malignant cases except the ANC. The LIT level was 204.34 ± 66.97 in the control group and 269.67 ± 145.16 (p < 0.05) in the cancer group (**Table 1**). Among the patients in the cancer group, the number of cases of T0 to T4 stage was 18 (22.78%), 21 (26.58%), 17 (21.52%), 12 (15.19%), and 11 (13.92%), respectively. A total of 55 patients (68.75%) of the malignant cases were at the N0 stage, and 76 (95%) were at the M0 stage. The most prevalent malignant tumor type in our study was cervical cancer (19.51%) (**Table 2**).

**Table 1:** Patient demographics, laboratory characteristics, and LIT scores.

Parameter Mean ± SD, n (%)	Benign (n = 79)	Malignant (n = 82)	Total (n = 161)
Age (year)	48.81 ± 13.42	42.99 ± 10.91	45.94 ± 12.55
BMI (kg/m^2^)	23.60 ± 3.44	23.35 ± 3.13	23.48 ± 3.28
RBC (10^12^/L)	4.02 ± 0.56	4.06 ± 0.60	4.04 ± 0.58
WBC (10^9^/L)	6.49 ± 2.22	6.91 ± 2.51	6.70 ± 2.37
Hemoglobin (g/L)	114.85 ± 16.95	118.05 ± 24.69	116.46 ± 21.20
Platelets (10^9^/L)	256.91 ± 73.94	235.94 ± 74.63	246.29 ± 74.79
ANC (10^9^/L) *	3.76 (1.45–10.01)	4.35 (1.45–5,096)^#^	3.915 (1.45–5,096)^#^
ALC (10^9^/L)	1.78 ± 0.70	1.84 ± 2.47	1.81 ± 1.80
NLR	2.11 (0.73–15.06)	2.78 (0.79–3,107.32)^#^	2.35 (0.73–3,107.32)^#^
ESR (mm/h)	26.83 ± 28.04	13.53 ± 17.43	16.72 ± 20.62
CRP (µg/ml)	21.42 ± 29.08	31.00 ± 25.29	27.34 ± 26.79
PCT (pg/ml)	1.12 ± 1.17	2.36 ± 2.17	2.18 ± 2.09
D-dimer (μg/ml)	1.13 ± 2.08	0.81 ± 1.28	0.97 ± 1.71
CEA (ng/ml)	3.96 ± 11.91	1.20 ± 0.65	2.44 ± 8.06
AFP (ng/ml)	2.29 ± 1.00	2.60 ± 1.13	2.43 ± 1.07
T-PSA (ng/ml)	0.78 ± 1.40	1.29 ± 1.73	0.96 ± 1.51
F-PSA (ng/ml)	0.16 ± 0.42	0.22 ± 0.31	0.18 ± 0.38
HCG (mIU/ml)	32.82 ± 166.73	0.66 ± 0.27	22.92 ± 138.73
MCCL (mm)	51.54 ± 28.56	33.55 ± 26.21	43.44 ± 28.84
LIT*	204.34 ± 66.97	269.67 ± 145.16	237.83 ± 118.24
Gender			
F	74 (93.6)	61 (74.39)	135 (83.85)
M	5 (6.33)	21 (25.61)	26 (16.15)
Smoking test			
No	75 (94.94)	65 (81.25)	140 (88.05)
Yes	4 (5.06)	15 (18.75)	19 (11.95)

BMI, body mass index; RBC, red blood cell; WBC, white blood cell; ANC, absolute neutrophil count; ALC, absolute lymphocyte count; NLR, neutrophil-to-lymphocyte ratio; ESR, erythrocyte sedimentation rate; CRP, C-reactive protein; PCT, procalcitonin; CEA, carcinoembryonic antigen; AFP, alpha-fetoprotein; PSA, prostate-specific antigen; HCG, human chorionic gonadotropin; MCCL, maximum cancer core length; LIT, Leukocyte ImmunoTest.

*p < 0.05 (p-value for ANC = 0.0185, p-value for LIT = 0.0188).

^#^The large variation was due to one patient whose ANC and NLR were extremely high; therefore, both are presented as median (min–max).

**Table 2:** Patients’ tumor-related data.

Parameter Mean ± SD, n (%)	Benign (n = 79)	Malignant (n = 82)	Total (n = 161)
Tumor stage (T)			
T0	20 (25.64)	18 (22.78)	38 (24.20)
T1	32 (41.03)	21 (26.58)	53 (33.76)
T2	17 (21.79)	17 (21.52)	34 (21.66)
T3	7 (8.97)	12 (15.19)	19 (12.10)
T4	2 (2.56)	11 (13.92)	13 (8.28)
Lymph node stage (N)			
N0	77 (98.72)	55 (68.75)	132 (83.54)
N1	1 (1.28)	24 (30.00)	25 (15.82)
N3	0 (0)	1 (1.25)	1 (0.63)
Metastasis stage (M)			
M0	78 (100.00)	76 (95.00)	154 (97.47)
M1	0 (0)	4 (5.00)	4 (2.53)
Cancer type			
Adenomyosis	1 (1.27)	0 (0)	1 (0.62)
Adrenal adenoma	0 (0)	4 (4.88)	4 (2.48)
Benign breast lumps	17 (21.52)	0 (0)	17 (10.56)
Benign lung tumor	2 (2.53)	0 (0)	2 (1.24)
Benign thyroid nodule	4 (5.06)	0 (0)	4 (2.48)
Breast cancer	0 (0)	7 (8.54)	7 (4.35)
Cervical cancer	0 (0)	16 (19.51)	16 (9.94)
Colorectal cancer	0 (0)	7 (8.54)	7 (4.35)
Endometrial cancer	0 (0)	6 (7.32)	6 (3.73)
Gastric cancer	0 (0)	9 (10.98)	9 (5.59)
Hysteromyoma	10 (12.66)	0 (0)	10 (6.21)
Kidney cancer	0 (0)	1 (1.22)	1 (0.62)
Lung cancer	0 (0)	1 (1.22)	1 (0.62)
Lung lesion	1 (1.27)	0 (0)	1 (0.62)
Mature teratoma ovarian tumor	0 (0)	1 (1.22)	1 (0.62)
Mediastinal cyst	1 (1.27)	0 (0)	1 (0.62)
Metropolypus	1 (1.27)	0 (0)	1 (0.62)
Non-small cell lung cancer	0 (0)	1 (1.22)	1 (0.62)
Esophageal cancer	0 (0)	2 (2.44)	2 (1.24)
Ovarian tumor	0 (0)	6 (7.32)	6 (3.73)
Rectal cancer	0 (0)	1 (1.22)	1 (0.62)
Small round cell tumor	0 (0)	1 (1.22)	1 (0.62)
Teratoma	2 (2.53)	0 (0)	2 (1.24)
Thoracic tumor	1 (1.27)	0 (0)	1 (0.62)
Thyroid cancer	0 (0)	19 (23.17)	19 (11.80)
Uterine fibroid	39 (49.37)	0 (0)	39 (24.22)

### Univariate and multivariate analyses of Leukocyte ImmunoTest-correlated clinical outcomes

Univariate analysis to investigate the association between patients’ clinical or tumor-related characteristics and LIT level showed that LIT level was associated with age (p < 0.05), smoking status (p < 0.001), white blood cell count (p < 0.001), CEA level (p < 0.001), AFP level (p < 0.01) (**Table 3**), tumor stage T4 (p < 0.001), lymph node stage N1 (p < 0.01), and tumor types (**Table 4**). Patients with kidney cancer (p < 0.05), rectal cancer (p < 0.01), and small round cell tumor (p < 0.01) showed higher LIT levels (**Table 4**).

**Table 3:** Univariate analysis of correlation between clinical variables and LIT score.

Variable	n	Coefficient	Adjusted R^2^	p-Value
Age	151	1.687	0.025	<0.05
BMI	129	−1.428	−0.006	NS
RBC	154	−5.910	−0.006	NS
WBC	154	18.518	0.129	<0.001
Hemoglobin	154	−0.096	−0.006	NS
Platelets	153	0.120	−0.001	NS
Neutrophil	152	−0.011	−0.005	NS
Lymphocyte	150	1.047	−0.007	NS
NLR	150	−0.018	−0.005	NS
ESR	25	1.601	0.086	NS
CRP	32	1.124	−0.006	NS
PCT	28	27.127	0.055	NS
D-dimer	104	13.819	0.027	NS
CEA	96	5.750	0.102	<0.001
AFP	59	54.725	0.134	<0.01
T-PSA	32	−0.981	−0.033	NS
F-PSA	32	−10.498	−0.031	NS
HCG	39	−0.004	−0.027	NS
Smoking	156	103.117	0.076	<0.001

BMI, body mass index; RBC, red blood cell; WBC, white blood cell; NLR, neutrophil-to-lymphocyte ratio; ESR, erythrocyte sedimentation rate; CRP, C-reactive protein; PCT, procalcitonin; CEA, carcinoembryonic antigen; AFP, alpha-fetoprotein; PSA, prostate-specific antigen; HCG, human chorionic gonadotropin; LIT, Leukocyte ImmunoTest.

**Table 4:** Univariate analysis of correlation between tumor-related variables and LIT score.

Variable	n	Coefficient	Adjusted R^2^	p-Value
Tumor stage (T)	154	NA	0.074	NA
T1		32.878		NS
T2		41.459		NS
T3		−19.690		NS
T4		131.253		<0.001
Lymph node stage (N)	155	NA	0.046	NA
N1		78.677		<0.01
N3		40.177		NS
Metastasis stage (M) M1	155	85.569	0.007	NS
MCCL	109	−0.183	−0.008	NS
Tumor type	158	NA	0.156	NA
Adrenal adenoma		87.500		NS
Breast cancer		109.286		NS
Cervical cancer		77.100		NS
Colorectal cancer		174.643		NS
Endometrial cancer		121.583		NS
Gastric cancer		209.667		NS
Kidney cancer		312.000		<0.05
Lung cancer		−30.500		NS
Non-small cell lung cancer		67.500		NS
Esophageal cancer		132.250		NS
Ovarian tumor		112.000		NS
Rectal cancer		447.000		<0.01
Small round cell tumor		473.500		<0.01
Thoracic tumor		28.000		NS
Thyroid cancer		101.105		NS

MCCL, maximum cancer core length; LIT, Leukocyte ImmunoTest.

**Figure 1:**
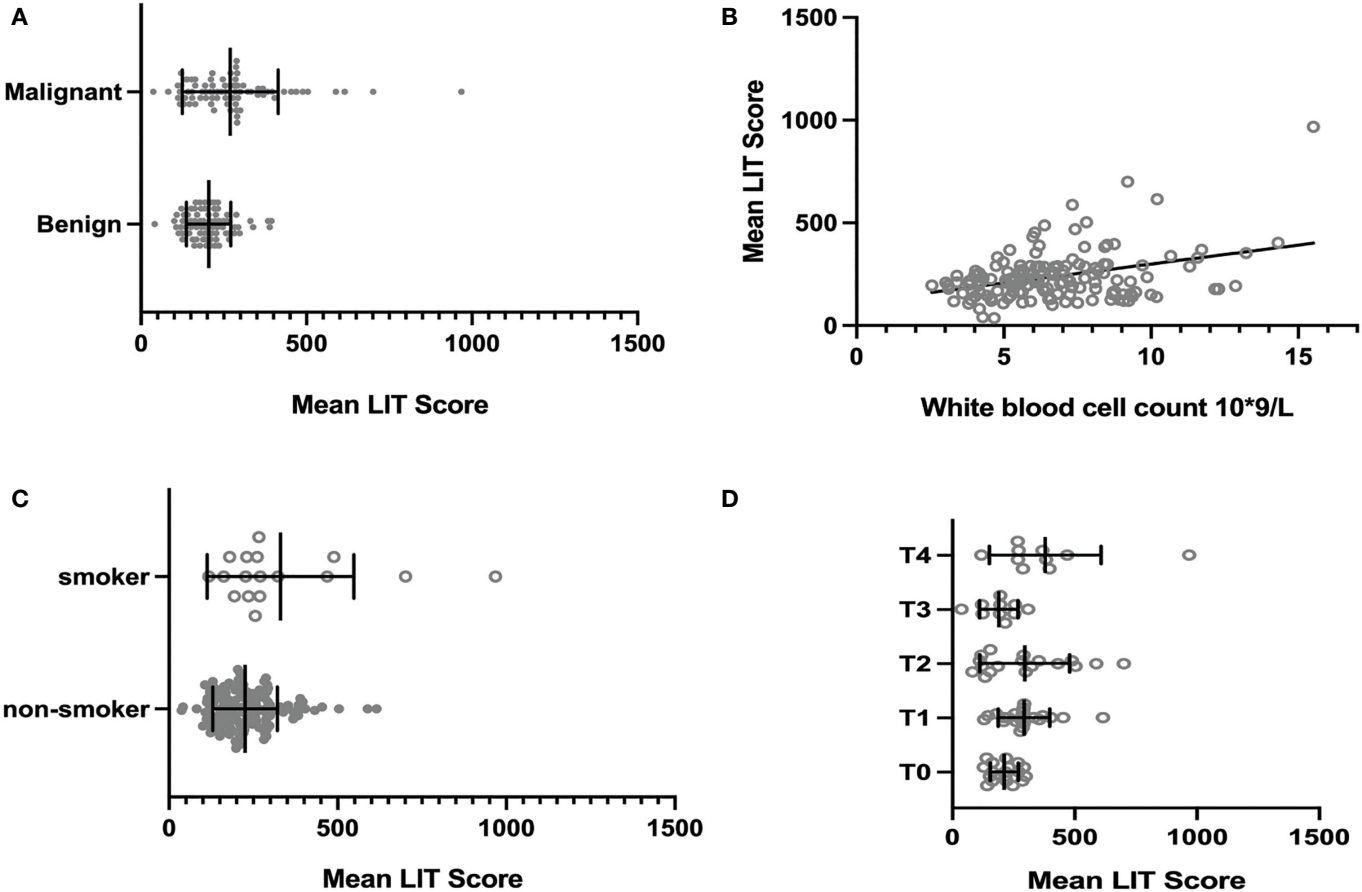
The association between all patients’ clinical or tumor-related characteristics and their Leukocyte ImmunoTest (LIT) score. **(A)** The mean LIT score of cancer group and control group. The mean LIT score of cancer group was significantly higher than that of control group (p-value = 0.0004). **(B)** The correlation between mean LIT score and white blood cell count. The mean LIT score and white blood cell count were positively correlated in all patients (p-value < 0.05). **(C)** The mean LIT score of smokers and non-smokers. The mean LIT score of patients who are smokers was significantly higher than that of non-smokers (p-value < 0.01). **(D)** The mean LIT score of patients with different tumor stages. The mean LIT score of patients with T4 stage tumors is significantly higher than that of patients with other tumor stages (p-value < 0.05).

**Figure 2:**
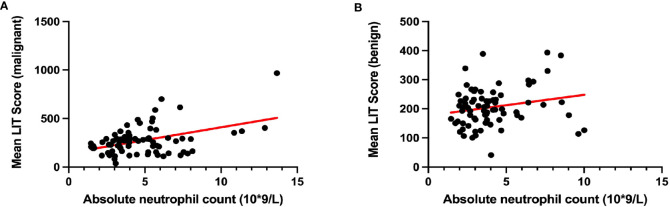
The correlation between patients’ Leukocyte ImmunoTest (LIT) score and their absolute neutrophil count. **(A)** The correlation between LIT score and absolute neutrophil count in cancer group. **(B)** The correlation between LIT score and absolute neutrophil count in control group. Pearson’s correlation test indicated that mean LIT score and absolute neutrophil count were positively correlated in patients with malignant tumor (**A**; Pearson’s r = 0.4329 [0.2299, 0.5998], p-value < 0.0001), but not in patients with benign tumor (**B**; Pearson’s r = 0.2102 [−0.01917, 0.4186], p-value = 0.0722).

To reduce type 1 errors, multivariate analysis was then carried out, and model fit was compared by the R^2^. Significant positive correlations were observed between LIT and tumor being malignant or benign (p < 0.001, **Figure 1A**), white blood cell count (p < 0.05, **Figure 1B**), smoking status (p < 0.01, **Figure 1C**), and tumor stage T4 (p < 0.05, **Figure 1D**) among all patients (**Table 5**). Pearson’s correlation test showed that the LIT score was positively correlated with the ANC in the cancer group (r = 0.4329 [0.2299, 0.5998], p-value < 0.0001), but not correlated in the control group (r = 0.2102 [−0.01917, 0.4186], p-value = 0.0722) (**Figure 2**).

**Table 5:** Multivariate analysis of correlation between tumor being malignant or benign, white blood cell count, smoking status, tumor stage, and LIT score.

Variable	Coefficient	95% CI	p-Value
Tumor being malignant or benign	42.277	7.842–76.711	<0.001
WBC	16.090	8.996–23.184	<0.05
Smoking status	70.238	18.226–122.249	<0.01
T4	64.901	3.199–146.893	<0.05

LIT, Leukocyte ImmunoTest; WBC, white blood cell.

### Leukocyte ImmunoTest: A potential diagnostic approach for cancer

**Figure 3:**
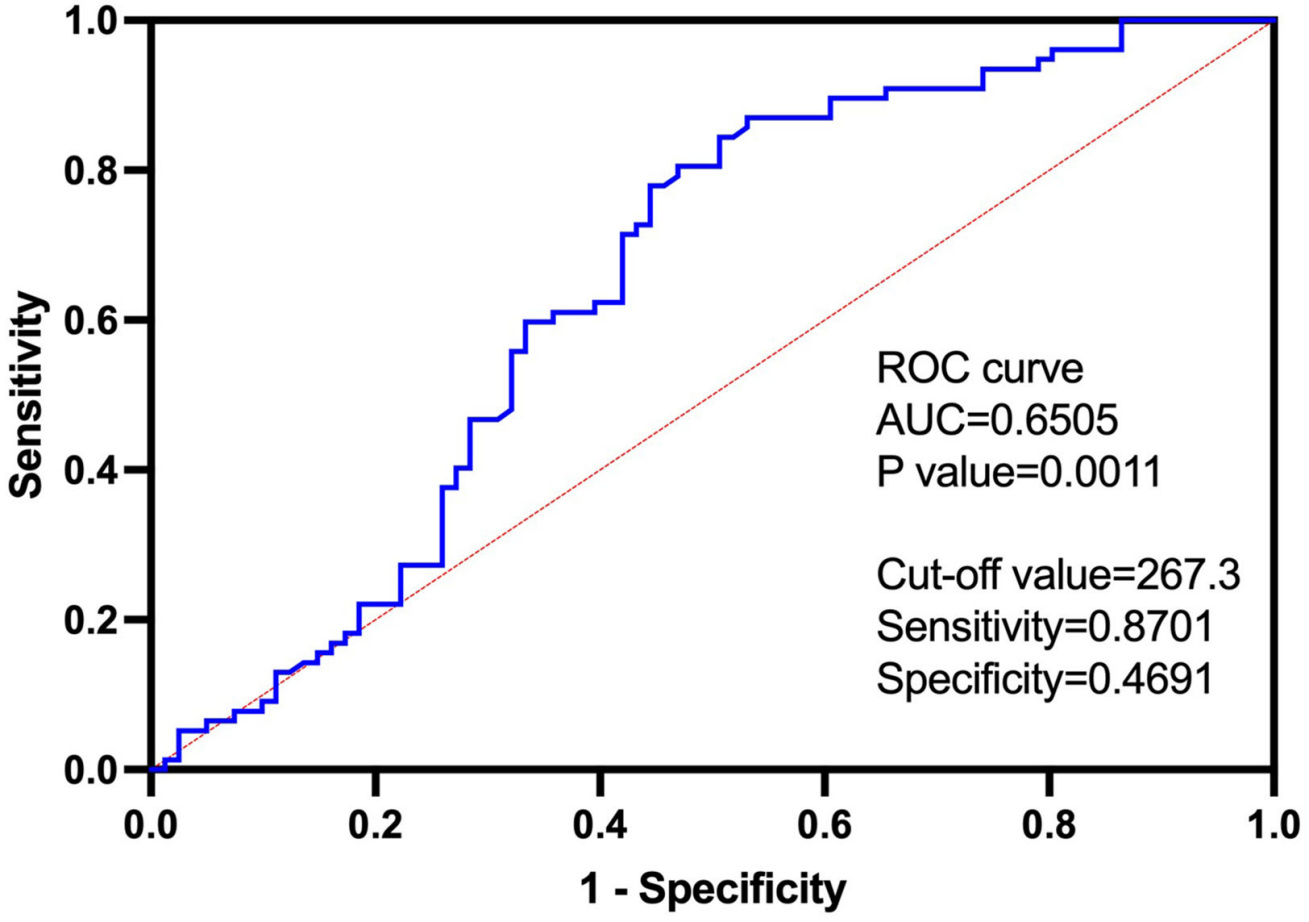
The receiver operating characteristic (ROC) curve of Leukocyte ImmunoTest (LIT) in predicting malignant tumor, with cutoff value of 267.3, sensitivity of 0.8701, and specificity of 0.4691. The area under the curve (AUC) was 0.6505. Positive predictive value of LIT on tumor being malignant is 63%, and negative predictive value is 84.7%.

The ROC curve demonstrated a significant diagnostic potential (area under the curve (AUC) = 0.6505, p-value = 0.0011) of LIT to identify malignant tumors and benign tumors (**Figure 3**). When the LIT cutoff value was set at 267.3, the sensitivity and specificity were 0.8701 (87.01%) and 0.4691 (46.91%), respectively (**Figure 3**). The positive predictive value of LIT on tumors being malignant was 63%, and the negative predictive value was 84.7%. The sensitivity and specificity of other diagnostic markers used in this study including inflammatory markers and tumor markers are presented in **Table 6**. PCT presented the highest level of sensitivity (100%) in cancer diagnosis in the studied population, followed by F-PSA (90.48%) and LIT (87.01%). In general, tumor markers and LIT showed better sensitivity as compared to inflammatory markers. However, the markers with good sensitivity such as PCT, F-PSA, and LIT showed a poor-to-moderate level of specificity. D-dimer had the highest specificity (83.64%) in cancer diagnosis, but the sensitivity of D-dimer was only 31.37%.

**Table 6:** Sensitivity and specificity of LIT and conventional cancer diagnostic markers.

	Variable	Cutoff value	Sensitivity	Specificity
Inflammatory marker	NLR	2.18	0.6104	0.7105
	ESR	14.50	0.6667	0.7895
	CRP	23.05	0.7692	0.6667
	PCT	2.570	1	0.2917
	D-dimer	0.9050	0.3137	0.8364
Tumor Marker	CEA	1.405	0.75	0.4783
	AFP	1.860	0.6061	0.7857
	T-PSA	0.61	0.7619	0.7273
	F-PSA	0.095	0.9048	0.4545
	HCG	0.94	0.3704	0.8333
	LIT	267.3	0.8701	0.4691

NLR, neutrophil-to-lymphocyte ratio; ESR, erythrocyte sedimentation rate; CRP, C-reactive protein; PCT, procalcitonin; CEA, carcinoembryonic antigen; AFP, alpha-fetoprotein; PSA, prostate-specific antigen; HCG, human chorionic gonadotropin; LIT, Leukocyte ImmunoTest.

## Discussion

Our study found that a significantly high level of the LIT was associated with malignant tumors; in particular, those patients who were in the advanced T4 tumor stage had high readings of LIT. The LIT level also showed a positive correlation with ANC in cancer patients. Further data analysis showed that LIT may be a predicting factor for tumor being malignant or benign with the cutoff value of LIT of 267.3.

LIT test was initially developed by McLaren et al., measuring the activity change of leukocytes (mainly, but not limited to neutrophils) as a bio-indicator for mental stress load (8). This technique is based on detecting PMA-induced burst production of ROS from circulating leukocytes, primarily neutrophils. LIT luminometer measured the emission of photons released during the interaction between ROS and luminol. In our study, the ability of leukocytes to react to PMA stimulation and release ROS was likely affected by the tumor environment (such as tumor-derived cytokines) per se. It is likely that patients with advanced tumors tend to have more tumor-activated leukocytes, favoring the production of higher levels of PMA-induced ROS during the LIT test (**Figures 1D** and **3**).

It is well known that immune cells work as the first-line defense against external molecules including infectious pathogens and early-stage tumors in the host. Nevertheless, immune cells tend to have a multifaceted function in tumor development due to tumor-induced phenotype alteration. Despite that, the role of immune cells in tumorigenesis remains controversial (12, 13), with a large variety of pro-tumoral leukocytes being recruited to tumor sites *via* tumor-released cytokines and chemokines. For instance, an increased level of infiltrated tumor-associated macrophages is found around tumors, promoting tumor progression by enhancing vascular endothelial growth factor A-mediated angiogenesis (14) and suppressing cytotoxic T cell-regulated anti-tumor response (15). Another good example is tumor-associated neutrophils (TANs), which are largely recruited and activated by Ras-regulated CXC chemokines in the tumor microenvironment (16). Previous studies also demonstrated increased TANs in patients with lung cancer, melanoma, head and neck cancer, pancreas cancer, prostate cancer, and breast cancer (17–23). In line with these studies, our data (**Table 1**) also suggested significantly higher levels of ANC in the cancer group compared to the control group. Moreover, a positive correlation between ANC and LIT was only found in the cancer group but not in the control group. This indicates that neutrophils in cancer patients were more susceptible to PMA stimulation than those in non-cancer patients. Despite the anti-tumoral roles of TANs, accumulated TANs are often linked with poor overall survival, recurrence-free survival, and disease-specific survival outcomes (24). Patients at the T4 stage had highly activated leukocytes (more capable of producing ROS with external stimulation). These cells facilitated cancer cells outgrowth and invasion *via* extracellular matrix remodeling and immunosuppression (25, 26). As the TNM stage is clinically used to assess cancer prognosis, the strong correlation between LIT and the TNM stage may suggest the potential prognostic value of LIT.

In our univariate analysis, patients’ age, smoking status, WBC count, CEA level, AFP level, tumor being malignant or benign, tumor stage T4, lymph node stage N1, and specific tumor types showed a strong association with LIT. However, multivariate analysis assisted to exclude any significance caused by interaction between variables, identifying only tumors being malignant or benign, WBC count, smoking status, and tumor stage affected LIT in all patients in our study. Particularly, ANC data showed a positive correlation with LIT in cancer patients but not in controls. It is worth pointing out that we found large variations of ANC and NLR in the cancer group (**Table 1**). This was due to one single patient whose ANC is 5,096 × 10^9^/L, leading to a large sample variation. This patient was with T3 esophageal cancer and was also a smoker. Esophageal cancer presents very high mortality that contributes to 5.3% of all cancer-related deaths (27). Patients with advantage esophageal cancer usually show poor outcomes with 5-year survival between 15% and 20% (28). It is known that immune cells show a sensitive response to tumor growth and act as potential prognostic markers (29). The high neutrophil level has been reported to be an independent factor for the poor prognosis of esophageal cancer (30). Several studies suggested the association of high NLR with worse overall survival in esophageal cancer (31–33). Furthermore, previous studies demonstrated that the number of peripheral blood neutrophils significantly increased after smoking (34, 35). Although this patient’s ANC was extremely high, all patients’ data were included for further analysis to reflect the nature of the proof-of-concept study. However, follow-up of this patient and further study may assist us to understand the cancer biology of patients with extremely high neutrophil levels.

Apart from identifying the predictive value of LIT in cancer diagnosis, we also compared the sensitivity and specificity between LIT and other diagnostic markers. Our data indicate relatively high sensitivity (87.01%) and moderate specificity (46.91%) of LIT in the studied population. The moderate specificity of LIT might be due to the choice of the control group, who were patients with benign tumors rather than healthy volunteers. Benign tumors, especially sizable benign tumors, usually exist with chronic inflammation and can also trigger complex immune responses (36). Thus, the immune status of the control group and cancer group might share a certain level of similarity and lead to decreased specificity for cancer detection in this study. We noticed that inflammatory marker PCT presented an extremely high level of sensitivity (100%) and an extremely low level of specificity (29.17%) in our study. PCT is one of the most sensitive parameters in detecting bacteria-associated infections. For cancer patients with infections, the PCT concentration may therefore be increased regardless of the existence of a malignant tumor, leading to poor sensitivity for cancer (37). Compared to inflammatory markers, tumor markers including CEA, AFP, HCG, and PSA generally showed better sensitivity for cancers. However, there are concerns about the accuracy of these tumor markers as well. For example, the positive serum concentration of CEA varies dramatically among colorectal cancer, pancreatic cancer, and breast cancer (38). A high level of CEA was reported in various non-cancer conditions such as hepatitis, alcoholic cirrhosis, and ulcerative colitis (38).

There were limitations to this study. For instance, LIT is highly associated with leukocyte activity, which is known to be susceptible to various factors. In our study, patients with immune function disorders and infections that may influence LIT reading were excluded to minimize confounding factors, but other factors that may affect our data remain unknown. For example, smoking is a factor found in this study that was unexpected, although it is well known that cigarette smoking triggers inflammatory responses including increased WBC count and inflammatory mediators (39). A relatively small sample size may not represent patients’ generality. Lastly, despite patient recruitment being random, more female patients were involved in this study, leading to gender imbalance. All these warrant further studies to verify the value of LIT for clinical use and to justify its several technical advantages: i) no centrifugation or plating of blood cells is required, preventing procedure-induced leukocyte activation; ii) a relatively small amount of blood is needed, and the device is portable, making this test easily accessible; iii) chemiluminometer reading data can be given within 10 min, reducing cost and waiting time.

In conclusion, this proof-of-concept study proposes the potential value of LIT in rapidly identifying whether a tumor is malignant or benign, as a predictor to be considered by clinicians and pathologists. Compared to biopsy or routine imaging approaches, LIT is a non-invasive, low-cost, and rapid measurement for cancer diagnosis. Nevertheless, larger sample sizes and cancer type-specific clinical studies are required to further evaluate the diagnostic value of LIT. A long-term follow-up study is also needed to assess the prognostic potential of LIT.

## Data availability statement

The raw data supporting the conclusions of this article will be made available by the authors, without undue reservation.

## Ethics statement

The studies involving human participants were reviewed and approved by Ethics Committee of Henan Provincial People’s Hospital, Zhengzhou University, Zhengzhou, China. The patients/participants provided their written informed consent to participate in this study.

## Author contributions

XL coordinated the study and contributed to the experimental design, interpretation of results, and writing of the paper. XM conducted a study on-site, patient recruitment, and data collection. YL contributed to the interpretation of the results. EC contributed to patient recruitment. JC provided study support and writing of the paper. JZ, DM and XL conceived the study design, supervised the project, and drafted and corrected the manuscript. All authors contributed to the article and approved the submitted version.

## Funding

The study was supported by Seroxo Ltd. The company also supplied the regents and monitor free of charge. The funder had no role in study design, data collection and analyses, and manuscript preparation for publication. JZ’s current research was supported by the Natural Science Foundation (No. 82071217, 81771149), Beijing, China, and the Department of Science and Technology (No. GZS2021005, 202102310124), Henan Province, China. Prof. Ma’s current research was supported by a grant from the National Institute of Health Research and *British Journal of Anaesthesia*, London, UK, and, the *European Society of Anaesthesiology and Intensive Care* (*ESAIC*), Brussels, Belgium.

## Acknowledgments

We thank Seroxo Ltd. for its generosity in supporting this research.

## Conflict of interest

The authors declare that the research was conducted in the absence of any commercial or financial relationships that could be construed as a potential conflict of interest.

## Publisher’s note

All claims expressed in this article are solely those of the authors and do not necessarily represent those of their affiliated organizations, or those of the publisher, the editors and the reviewers. Any product that may be evaluated in this article, or claim that may be made by its manufacturer, is not guaranteed or endorsed by the publisher.

## Abbreviations

AFP, alpha-fetoprotein; ALC, absolute lymphocyte count; ANC, absolute neutrophil count; AUC, area under the curve; BMI, body mass index; CEA, carcinoembryonic antigen; CRP, C-reactive protein; ESR, erythrocyte sedimentation rate; F-PSA, free prostate-specific antigen; HCG, human chorionic gonadotropin; LIT, Leukocyte ImmuneTest; MCCL, maximum cancer core length; NLR, neutrophil-to-lymphocyte ratio; PCT, procalcitonin; PMA, phorbol 12-myristate 13-acetate; RBC, red blood cell; ROC, receiver operating characteristic; ROS, reactive oxygen species; TANs, tumor-associated neutrophils; TNM, tumor-node-metastases; T-PSA, total prostate-specific antigen; WBC, white blood cell.
